# Cross-sectional examination of the association between shift length and hospital nurses job satisfaction and nurse reported quality measures

**DOI:** 10.1186/s12912-017-0221-7

**Published:** 2017-05-25

**Authors:** Jane Ball, Tina Day, Trevor Murrells, Chiara Dall’Ora, Anne Marie Rafferty, Peter Griffiths, Jill Maben

**Affiliations:** 1 National Institute for Health Research Collaboration for Leadership in Applied Health Research and Care (NIHR CLAHRC), Wessex, Southampton, UK; 2 University of Southampton, Southampton, UK; 3 Medical Management Centre (MMC), Department of Learning, Informatics, Management and Ethics (LIME), Karolinska Institutet (KI), Stockholm, Sweden; 4 Florence Nightingale Faculty of Nursing and Midwifery, King’s College London, James Clerk Maxwell Building, 57 Waterloo Road, London, SE1 8WA UK

**Keywords:** Shift work, 12 h shift, Work hours, Care left undone, Quality of health care, Job satisfaction, Patient safety, England

## Abstract

**Background:**

Twenty-four hour nursing care involves shift work including 12-h shifts. England is unusual in deploying a mix of shift patterns. International evidence on the effects of such shifts is growing. A secondary analysis of data collected in England exploring outcomes with 12-h shifts examined the association between shift length, job satisfaction, scheduling flexibility, care quality, patient safety, and care left undone.

**Methods:**

Data were collected from a questionnaire survey of nurses in a sample of English hospitals, conducted as part of the RN4CAST study, an EU 7^th^ Framework funded study. The sample comprised 31 NHS acute hospital Trusts from 401 wards, in 46 acute hospital sites. Descriptive analysis included frequencies, percentages and mean scores by shift length, working beyond contracted hours and day or night shift. Multi-level regression models established statistical associations between shift length and nurse self-reported measures.

**Results:**

Seventy-four percent (1898) of nurses worked a day shift and 26% (670) a night shift. Most Trusts had a mixture of shifts lengths. Self-reported quality of care was higher amongst nurses working ≤8 h (15.9%) compared to those working longer hours (20.0 to 21.1%). The odds of poor quality care were 1.64 times higher for nurses working ≥12 h (OR = 1.64, 95% CI 1.18–2.28, *p* = 0.003).

Mean ‘care left undone’ scores varied by shift length: 3.85 (≤8 h), 3.72 (8.01–10.00 h), 3.80 (10.01–11.99 h) and were highest amongst those working ≥12 h (4.23) (*p* < 0.001). The rate of care left undone was 1.13 times higher for nurses working ≥12 h (RR = 1.13, 95% CI 1.06–1.20, *p* < 0.001).

Job dissatisfaction was higher the longer the shift length: 42.9% (≥12 h (OR = 1.51, 95% CI 1.17–1.95, *p* = .001); 35.1% (≤8 h) 45.0% (8.01–10.00 h), 39.5% (10.01–11.99 h).

**Conclusions:**

Our findings add to the growing international body of evidence reporting that ≥12 shifts are associated with poor ratings of quality of care and higher rates of care left undone. Future research should focus on how 12-h shifts can be optimised to minimise potential risks.

## Background

This study was a secondary analysis of data collected in England as part of the RN4Cast study, exploring the risk of negative outcomes with nurses working 12 h shifts. Specifically, we sought to establish whether there was an association between shift length and reported outcomes: nurse job satisfaction, satisfaction with work flexibility, care quality, patient safety, and care left undone. This paper is based on a report published to the research funder, NHS England [1]. This report is available online via the web, but was neither peer reviewed nor widely disseminated and should be viewed as a report to the funder and not an academic publication.

The provision of 24-h nursing care involves shift work, including “long days” or 12-h shifts [2, 3]. Historically, shift patterns were based on three eight-hour shifts per day [4, 5] but over the past 20 years there has been a tendency to move towards the 12-h shift [6, 7]. In the last few decades, an increasing number of NHS hospitals in England started to utilise 12-h shifts in the belief that it is a more cost effective way of providing 24-h care, with fewer overlaps between shifts, offering greater continuity of staffing over day and night [8]. However, claims of financial benefits of 12-h shifts by NHS Trust Boards are made in the absence of economic evaluations. Furthermore, some nurses prefer to work longer daily hours with fewer shifts, giving them greater flexibility and more days away from work [9–11]. As the majority of the nursing workforce is female, this may also make it easier to balance work and personal responsibilities but long days may carry hidden costs for staff and patients [11, 12].

However, some employers are increasingly concerned over potential threats to patient safety and quality of care and are choosing to revert to eight-hour shifts [13, 14]. Although the handover period has been criticised for being unproductive, with no formal ‘overlap’, 12-h shifts can have a negative impact on opportunities for ward meetings, teaching, mentorship, teambuilding and research [15, 16]. A study by Stimpfel and colleagues found that nurses who worked shifts of 12-h or longer were significantly more likely to report poor quality care and poor patient safety when compared to those working eight-hour shifts [17]. Furthermore, a study including the patients’ perspective reported lower satisfaction with care in hospitals where staff worked longer shifts [18]. A recent systematic review of error rates among nurses found evidence of a higher risk of mistakes when working a 12 h shift compared to shorter shifts (most of the studies used 8 and 12 h as cut-off points) [19].

The shift length argument has been explored by other occupational sectors than nursing and experts believe that fatigue associated with long shifts played a major role in the unfolding of disasters such as the Chernobyl nuclear accident, Three Mile Island incident and the grounding of the Exxon Valdez [20] A systematic review by Smith and colleagues compared eight and 12-h shifts across a broad range of industries and concluded that working longer shifts without sufficient rest between shifts may increase fatigue and, therefore, pose a threat to safety [21]. However, research beyond health is equivocal and some studies have found little differences in terms of cost or productivity [22] or levels of fatigue [23] by shift length.

In nursing, Geiger-Brown and Trinkoff collated evidence on 12-h shifts and concluded that long shifts are unsafe for both patients, in terms of medication errors and for nurses, who are at greater risk of musculoskeletal diseases, needle stick injuries and drowsy driving behaviour [13]. Estabrooks and colleagues reviewed 12 studies comparing the effect of eight and 12-h shifts on quality of care and health care provider outcomes. They found insufficient evidence to conclude that shift length had an effect on patient or healthcare outcomes [4].

Two large European cross sectional studies of 31,627 registered nurses concluded that those working shifts of 12 h or longer were more likely to report poor quality of care, poor patient safety, and higher rates of care left undone [24] and higher levels of job dissatisfaction, burnout and intention to leave [25], when compared with nurses working 8 h or shorter shifts.

Harris et al. reviewed 85 studies published between 1973 and 2014 according to five broad themes: ‘risks to patients’, ‘patient experience’, ‘risks to staff’, ‘staff experience’ and ‘impact on organisational work’. The review concluded that the evidence of any clear effect of 12-h shifts is inconsistent in outcomes and study design [26].

Dall’Ora et al’s scoping review of the effect of shift work on employees’ performance and well-being synthesised shift patterns across all sectors, not just nursing. Although some large scale multicentre studies showed that 12 h shifts are associated with worse staff and patient outcomes, the authors concluded that most studies evaluated one single characteristic and failed to take account of the many complex facets of shift work. It was not therefore possible to draw firm conclusions as studies were often confounded by extraneous variables [27].

Current knowledge shows that widespread variation exists in shift length across the EU. Recent analysis of data from 12 EU countries (31,627 nurses in 2170 medical/surgical units within 487 hospitals) explored variation in the shift length nurses work between and within countries, and within hospitals [24]. Variation in typical shift length has been observed, with most countries presenting a clear 8 or 12 h shift pattern; England is unusual in presenting a mixed economy in shift patterns with 32% of day shifts and 36% of night shifts lasting 12 h or more making it a ‘natural laboratory’ for examining the effect of such variation on outcomes [24].

Data on nurses’ work patterns, including their working hours are not routinely collected in the UK. However, analysis of data collected through a series of cross-sectional surveys of nurses’ employment in the UK indicate that there has been a steep increase in the prevalence of nurses working long shifts (12-h plus) in NHS hospitals, from 31% in 2005 to 52% in 2009 [1].

## Methods

We used data from a survey of nurses in a random sample of English hospitals, conducted as part of the RN4Cast study, an EU 7^th^ Framework funded study of the nursing workforce covering 12 EU countries and three international partner countries beyond Europe [1]. The study sought to examine the relationship between nursing inputs and patient outcomes, whilst controlling for other potentially confounding factors. The study included a survey of registered nurses in medical and surgical wards in England. The sample comprised 31 NHS acute hospital Trusts (administrative groupings of hospitals) from 400 wards, in 46 acute hospital sites. The questionnaire covered: practice environment, staffing and patient numbers on the last shift worked, quality and safety measures, frequency of adverse events, care left undone, job dissatisfaction and working hours (including shift length). The survey was administered in spring/summer of 2010, 2917 registered nurses responded achieving an estimated response rate of 39%. Ethical approval for the RN4Cast study in England was sought and gained from the National Research Ethics Committee (Ref: 09/H0808/69) and permissions acquired for the research to be undertaken at each hospital. Informed consent was obtained from participants by completion of the questionnaire, as approved by the ethics committee.

### Measures

Five self-report measures representing care quality, safety and job and work schedule flexibility satisfaction were drawn from the survey. Four were converted into dichotomous (binomial) variables: poor quality of care nurse rating (*poor/fair*), poor patient safety rating (*failing/poor*), not satisfied with job (*very dissatisfied/a little dissatisfied*) and not satisfied with work schedule flexibility (*very dissatisfied/a little dissatisfied*). A fifth measure of care left undone was created from a list of 13 activities where respondents were asked: ‘On your most recent shift, which of the following activities were necessary but left undone because you lacked the time to complete them’. The number of activities left undone was counted to produce a score out of 13.

### Analysis

Descriptive analysis was undertaken, measures were described using frequencies, percentages and mean scores (care left undone with 95% confidence intervals) by shift length, working beyond contracted hours and day (including afternoon and evening) or night shift, and a box plot of shift length by day or night shift. Multi-level regression models were used to establish whether there were statistical associations between shift length and a number of nurse self-reported measures of care quality and job and work schedule flexibility satisfaction, whilst accounting for other factors and correcting for clustering within trusts and wards. The potential predictors identified were: shift length, working beyond contracted hours, day/night shift, medical or surgical unit, patients per nurse (grouped in patient increments of two), patients per HCA (Quintiles), full or part-time work, age (in ten year bands), Trust size, high (or not) technology trust, teaching (or non-teaching) trust.

A multilevel logistic model was fitted to each of the dichotomous measures, and a multilevel Poisson model to the number of activities left undone using IBM SPSS Version 22 GENLINMIXED. The dependent variables poor quality of care nurse rating (poor/fair), poor patient safety rating (failing/poor), not satisfied with job (very dissatisfied/a little dissatisfied with work schedule) were modelled assuming the data were generated from a binomial distribution. The care left undone score (thirteen items range 0–13) was modelled assuming the data were generated from a Poisson distribution.

Each model included random effects for intercepts at the ward and trust levels. These random effects help to establish whether significant residual variation remains between trusts and wards (within trusts) in the model, after the inclusion of the predictors, and to enable correct estimation of standard errors in the presence of clustering. It was not possible to fit random intercepts at both trust and ward levels for either poor quality of care nurse rating or poor patient safety rating. We dropped the trust level random intercept from the former and the ward level random intercept from the latter to achieve model convergence.

## Results

**Fig. 1:**
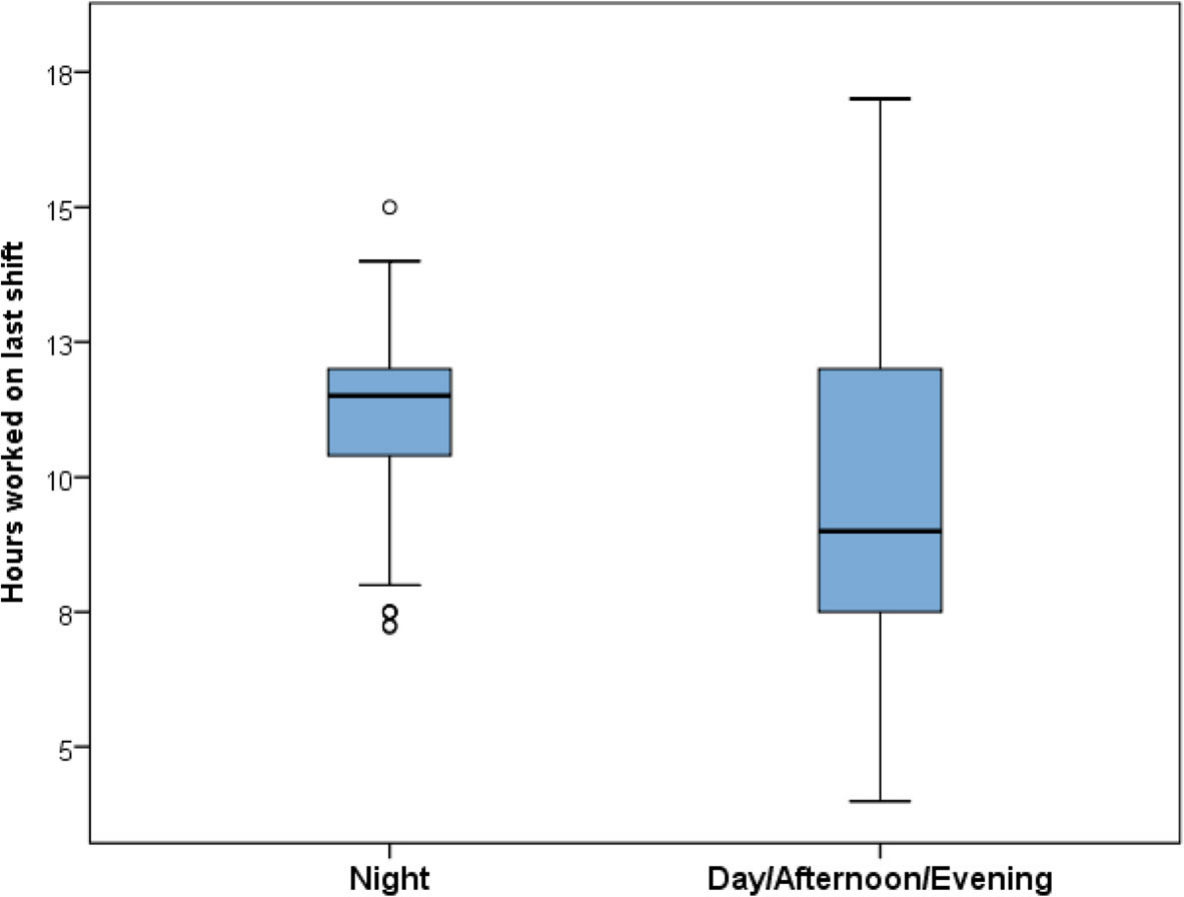
Shift length by day/night

A total of 2568 nurses (out of 2917) provided information on the length of their last shift and whether it took place during the day (morning/afternoon/evening) or at night of whom 74% (1898) had worked a day shift and 26% (670) a night shift (Table 1). Analysis at the ward level showed a high degree of variation in day shift durations between wards in the same hospitals; most Trusts having a mix of eight hour shifts, 12-h shifts, and shifts of a variety of other lengths. Few Trusts have a single shift length in operation across or within the wards studied (see Fig. 1).

**Table 1:** Descriptive statistics: quality of care, patient safety, care left undone, job satisfaction, work schedule flexibility by shifts

		Poor quality of nursing care rating		Poor patient safety rating		Care left undone		Not satisfied with job		Not satisfied with work schedule	
	No. in each category	No.	%	No.	%	Mean	(95% CI)	No.	%	No.	%
≤8 h shift	860	136	15.9	49	5.7	3.85	(3.72–3.98)	301	35.1	186	21.8
8.01–10.00	356	73	20.6	26	7.3	3.72	(3.52–3.92)	159	45.0	104	29.4
10.01–11.99	496	99	20.0	33	6.7	3.80	(3.63–3.98)	194	39.5	116	23.5
≥12	856	180	21.1	59	6.9	4.23	(4.09–4.37)	366	42.9	230	27.0
Not overtime	1269	198	15.7	62	4.9	3.24	(3.14–3.34)	412	32.7	253	20.0
Working beyond contracted hours	1289	288	22.5	105	8.2	4.67	(4.55–4.79)	605	47.1	380	29.6
Day shift	1898	340	18.0	116	6.1	4.11	(4.02–4.21)	730	38.5	434	23.0
Night shift	670	148	22.2	51	7.7	3.48	(3.34–3.62)	290	43.8	202	30.4

In Table 2 the results from the multi-level regression models are presented. The odds ratios (or in the case of care left undone the rate ratios) are given, with 95% confidence intervals comparing each shift length category (8.01–10.00 h, 10.01–11.99 h, ≥12 h) with shift length ≤ 8 h (reference category) adjusting for all other predictor variables.

**Table 2:** Multilevel regression models: associations between shift length and outcomes

	≤8 h shift (reference category)	8.01–10.00 h	10.01–11.99 h	≥12 h
Poor Quality of Nursing Care	1.00	1.21 (0.82–1.80)	1.43 (0.96–2.11)	1.64* (1.18–2.28)
Poor Patient Safety	1.00	1.00 (0.56–1.77)	0.99 (0.56–1.75)	1.17 (0.73–1.89)
Care Left Undone	1.00	0.97 (0.90–1.04)	1.05 (0.97–1.14)	1.13* (1.06–1.20)
Not satisfied with job	1.00	1.31 (0.97–1.77)	1.33 (0.98–1.80)	1.51* (1.17–1.95)
Not satisfied with work schedule	1.00	1.08 (0.78–1.51)	0.91 (0.64–1.28)	1.22 (0.92–1.61)

Controlling for: working beyond contracted hours, day/night shift, medical or surgical unit, patients per nurse (grouped in patient increments of two), patients per HCA (Quintiles), full or part-time work, age (in ten year bands), Trust size, high (or not) technology trust, teaching (or non-teaching) trust

Odds ratios (95% confidence interval); for the outcome Care left undone a rate ratio (RR) is provided instead

* Statistical significance *p* <0.01

The amount of self-reported poor quality of nursing care was lower amongst nurses working eight hours or less (15.9%) compared to those working longer hours (20.0 to 21.1%). Length of shift was significantly associated with poor quality of nursing care in the multilevel model (F[3,2314] = 2.95, *p* = .031). The odds of poor quality care was 1.64 times higher for nurses working a 12-h or longer shift compared to those working eight hours or less (OR = 1.64, 95% CI 1.18–2.28, *p* = 0.003).

A similar trend was apparent for safety ratings. A smaller proportion of those working shifts of eight hours or less rated patient safety as poor (5.7%) than nurses working a longer shift (6.7 to 7.3%). However, in the multi-level model, this relationship was not significant (F[3,2313] = 0.25, *p* = .86).

Mean ‘care left undone’ score varied by length of shift: 3.85 (≤8 h), 3.72 (8.01–10.00 h), 3.80 (10.01–11.99 h) and was highest amongst those working 12 h or over (4.23). This relationship was significant in the multi-level model (F[3,2326] = 6.37, *p* < 0.001). The rate of care left undone was 1.13 times higher for nurses working a 12 h or longer shift compared to those working eight hours or less (RR = 1.13, 95% CI 1.06–1.20, *p* < 0.001).

Nurse dissatisfaction with their job varied with length of shift: 35.1% (≤8 h shift), 45.0% (8.01–10.00 h), 39.5% (10.01–11.99 h) and 42.9% (≥12 h). This relationship was significant in the multilevel model (F[3,2318] = 3.46, *p* = .016). When taking the other predictor variables into account, the odds of being dissatisfied were 1.51 times higher for nurses working shifts of 12 h or more compared to those working eight hours or less (OR = 1.51, 95% CI 1.17–1.95, *p* = .001).

There was no clear pattern of variation in dissatisfaction with work schedule flexibility by length of shift: 21.8% (<8 h shift), 29.4% (8.01–10.00 h), 23.5% (10.01–11.99 h) and 27.0% (≥12 h) reported being dissatisfied. When this relationship was explored in multilevel model, the relationship was not significant (F[3,2314] = .1.45, *p* = .23).

## Discussion

England is unusual compared with other European countries in the diversity of shift lengths worked [24]. Our analysis of data from this cross-sectional study using multilevel regression models found that the length of shift worked by nurses was a predictor of care rated as ‘poor quality’. Working 12-h or longer shifts was significantly associated with a higher rate of necessary care being left undone. Nurses working 12-h shifts or longer reported higher levels of poor patient safety. However, when taking into account other factors (most notably staffing levels and working beyond the scheduled shift), shift length was not a statistically significant predictor of the overall patient safety rating of the ward.

Our findings add to the international body of evidence reporting that working 12 h shifts or longer are associated with poor ratings of quality of care and higher rates of care left undone [17, 24, 28, 29]. Some studies have proposed 12-h shifts as a way to improve efficiency [30], however our study indicates that these long work hours may compromise care. The premise that 12-h shifts are cost effective may be contested, as it is unlikely that a net increase in efficiency can be obtained while 12 h shifts are associated with an increase in care left undone with a consequent decline in care quality.

It may be reassuring to note that in our study there was no significant reduction in safety ratings, but other research points to contrasting conclusions, with a recent review describing evidence of the detrimental effects of long shifts on safety [25]. Furthermore, a US study of 633 nurses reported that inpatient deaths were significantly more likely to occur in hospitals where nurses reported schedules with long work hours [31]. If long shifts have previously shown to impact on mortality rates, more research needs to be done before concluding that 12-h shifts are safe or at least carry comparable risk to shorter shifts.

Anecdotally, nurses’ views of 12-h shifts are mixed; many are attracted by 12-h shifts as it compresses the working week into fewer days, allowing more time off and reducing travel time and costs, but some describe such shifts as exhausting and are concerned about the perceived adverse effect on performance [16, 29, 31, 32].

In our study, nurses working 12-h shifts were, however, no more or less satisfied with their work schedule flexibility than those working shorter shifts. However, our results highlight that when nurses are working 12-h shifts, they were less likely to express satisfaction with their jobs when compared to those working less than 12 h. The results reveal that when the samples of nurses are closely matched (all working in same type of ward in NHS acute trusts) and differences in the working context of nurses working long shifts are taken into account, nurses are less satisfied with their jobs compared to those working shifts of 8 h or less. This is in line with findings from the study by Dall’Ora et al. which also concludes that such long shifts are associated with job dissatisfaction. The authors hypothesise that “total life satisfaction” and “job satisfaction” are two different concepts and, therefore, even if nurses may find working fewer days appealing for their work-life balance, this shift pattern may lead to stress and fatigue on the job [25]. Individual nurses may hold a range of views on 12-h shifts including personal efficiency benefits in working longer shifts whilst nonetheless finding them very tiring and being concerned about the effects of fatigue on their ability to deliver good patient care.

### Limitations

This study relied on cross sectional and nurse-reported data, similar to the majority of shift length studies. This may have led to subjective interpretations of the outcome measures, for example some nurses may conceptualise “good quality of care” in a certain way that may not reflect the same concept for other nurses. The cross sectional nature of the data prevents us from inferring any cause-effect relationships between shift length and outcomes. Furthermore, most of the outcomes were captured by a single item question (e.g. In general, how would you describe the quality of nursing care delivered to patients on your unit/ward?). However, the whole picture is likely to be much more complex than a mean score can illuminate. Many different features of working patterns are identified as having a relationship with job performance [25]. However, in common with many of the studies in this field, this research has examined a single dimension - shift length and overtime– without taking into account other features such as shift sequences, breaks, rest time between shifts and control over working hours. More research is needed to understand how these features relate to one another, and the potential for positive working practices (such as sufficient rest times) to off-set the negative relationships reported here.

## Conclusions

The decision to introduce, keep, or remove the 12-h shift is a challenging one for nurse managers. From an employer’s point of view, a move to 12-h shifts can appear to reduce short term costs by reducing the overlap and enabling a reduction in workforce. But very little is known about either the long term effects on staff sickness absence and turnover or the effects of removing this period of overlap, which traditionally was a key time for learning and mentoring to take place for both staff and students. If 12-h shifts are associated with increased fatigue and more missed care then productivity can be lost. None of the studies reviewed included a review of these effects or provided economic evidence. More research is required in this area.

A key issue of 12-h shifts is that ‘it depends on how it’s done’. The question we have sought to address has been ‘what are the effects of working 12-h shifts?’ controlling for other factors. Future research should focus on how 12-h shifts be optimised to minimise the potential risks.

The analysis of data presented here raises a significant challenge to the assumption that 12-h shifts can reduce costs without any deleterious effects. In the absence of a more complete picture of both the effects and the costs of 12-h shifts, managers should proceed with caution.

## Abbreviations

95% CI: 95% confidence intervals
EU: Europe
HCA: Health Care Assistant
NHS: National Health Service
OR: Odds ratio
RN4CAST: Nurse forecasting in Europe
UK: United Kingdom
US: United States of America
